# IL-2 Inhibition of Th17 Generation Rather Than Induction of Treg Cells Is Impaired in Primary Sjögren’s Syndrome Patients

**DOI:** 10.3389/fimmu.2018.01755

**Published:** 2018-08-13

**Authors:** Jing Luo, Bingxia Ming, Cai Zhang, Xiaofei Deng, Pingfei Li, Zhengping Wei, Yu Xia, Kan Jiang, Hong Ye, Wanli Ma, Zheng Liu, Huabin Li, Xiang-Ping Yang, Lingli Dong

**Affiliations:** ^1^Department of Immunology, School of Basic Medicine, Tongji Medical School, Huazhong University of Science and Technology (HUST), Wuhan, China; ^2^Department of Rheumatology and Immunology, Tongji Hospital, Huazhong University of Science and Technology (HUST), Wuhan, China; ^3^Lymphocyte Cell Biology Section, National Institute of Arthritis and Musculoskeletal and Skin Diseases, National Institutes of Health, Bethesda, MD, United States; ^4^Department of Pathophysiology, School of Basic Medicine, Tongji Medical College, Huazhong University of Science and Technology (HUST), Wuhan, Hubei, China; ^5^Department of Respiratory and Critical Care Medicine, Union Hospital, Tongji Medical College, Huazhong University of Science and Technology (HUST), Wuhan, China; ^6^Department of Otolaryngology-Head and Neck Surgery, Tongji Hospital, Tongji Medical School, Huazhong University of Science and Technology (HUST), Wuhan, China; ^7^Department of Otolaryngology, Head and Neck Surgery, Affiliated Eye, Ear, Nose and Throat Hospital, Fudan University, Shanghai, China

**Keywords:** Sjögren syndrome, IL-2, Treg, Th17, p-STAT5

## Abstract

**Objective:**

To investigate the role of IL-2 in the balance of Th17 and Tregs and elucidate the underlying mechanisms of enhanced Th17 differentiation in primary Sjögren’s syndrome (pSS) patients.

**Methods:**

This study involved 31 pSS patients, 7 Sicca patients, and 31 healthy subjects. Th17 and Treg cells were determined by flow cytometry, and IL-17A was detected by immunohistochemistry. IL-2 and IL-6 levels were assessed by ELISA and qPCR. p-STAT5 and p-STAT3 in salivary glands (SGs) were evaluated by immunohistochemistry and flow cytometry. The binding of STAT5 and STAT3 to the *Il17a* gene locus was measured by chromatin immunoprecipitation.

**Results:**

We found that the percentage of Th17 cells was increased in the periphery and SG of pSS patients when compared with healthy subjects, but the Treg cells was unchanged. Meanwhile, the IL-2 level was reduced, and the IL-6 and IL-17A level was increased in the plasma of pSS patients. The ratio of IL-2 and IL-6 level was also decreased and IL-2 level was negatively correlated with the level of IL-17A. The expression of *Il6* and *Il17a* mRNA was significantly increased, whereas *Foxp3, Tgfb1, Tnfa*, and *Ifng* mRNA were comparable. Furthermore, the level of STAT5 phosphorylation (p-STAT5) was reduced and p-STAT3 was enhanced in the SGs and in peripheral CD4^+^ T cells of pSS patients. *In vitro* IL-2 treatment-induced STAT5 competed with STAT3 binding in human *Il17a* locus, leading to decreased Th17 differentiation, which was associated with the reduced transcription activation marker H3K4me3.

**Conclusion:**

Our findings demonstrated a Treg-independent upregulation of Th17 generation in pSS, which is likely due to a lack of IL-2-mediated suppression of Th17 differentiation. This study identified a novel mechanism of IL-2-mediated immune suppression in pSS.

## Introduction

Primary Sjögren’s syndrome (pSS) is a chronic autoimmune disease characterized by inflammatory infiltration of immune cells within lacrimal and salivary glands (SGs), which leads to the dysfunction of these exocrine glands (1–4). Besides B cells and CD8^+^ T cells, CD4^+^ T cells represent a significant population in the affected gland tissues (5).

Th17 cells are a lineage of pro-inflammatory CD4^+^ T cell subsets, which play critical roles in autoimmunity (6, 7). By contrast, FOXP3^+^ Treg cells are essential for mediating immune tolerance, and deficiency of Treg cells is often linked with autoimmunity (8). Th17 and Treg cells are reciprocally regulated by cytokines. For example, IL-6 favors Th17 differentiation but suppresses Treg generation (9, 10); conversely, IL-2 promotes Treg generation but inhibits Th17 differentiation (11, 12). In addition, we reported that IL-2-induced STAT5 activation limits Th17 differentiation and related autoimmunity in a murine model *via* competing the IL-6-induced STAT3 binding to the *Il17a/f* locus, in a FOXP3-independent fashion (13). However, whether IL-2-induced STAT5 activation limits human Th17 differentiation and plays a role in human autoimmune disease remains unclear.

Th17 cells and their associated cytokines are implicated in the pathogenesis of pSS (14–17). However, the roles of Treg cells in pSS are controversial. Liu and colleagues found reduced CD4^+^CD25^+^ Treg cells in the periphery of pSS (18), while another group found no reduction of Treg cells in pSS patients (19). Numerous clinical studies are investigating the therapeutic potential of IL-2 in autoimmune diseases and focus on the expansion of Tregs (20–23); however, it is not known whether the therapeutic efficacy of IL-2 is solely attributable to the expansion of Tregs. In addition to regulation of differentiation of multiple T cell lineages, IL-2 regulates T effector cell expansion, memory generation, and proliferation of NK cells and B cells (24–26). Inappropriate application of IL-2 can also exhibit high toxicity (27, 28). Thus, understanding the change of IL-2 level and its function in detail in pSS patients is essential for rational IL-2 therapeutic application.

In this study, we found an increased Th17 cells and unchanged Treg cells in pSS patients. The enhanced Th17 differentiation was associated with reduced IL-2 and p-STAT5 in pSS. Furthermore, treatment of IL-2 induced STAT5 competed with STAT3 for the binding to the *Il17a* locus, which directly suppressed Th17 differentiation but without perturbation of Treg differentiation. Our findings uncovered a direct signaling pathway of IL-2 which suppressed Th17 generation in a Treg cells independent manner in pSS.

## Materials and Methods

### Patients

31 pSS patients attending the Sjögren Clinic of Tongji Hospital of Huazhong University of Science and Technology were enrolled in this study. This study had the approval of the ethical committee of the Tongji Hospital and informed consent from every patient. The diagnosis of pSS was made according to the 2002 American-European Consensus Group criteria. Controls were either healthy subjects or patients with the Sicca syndrome. The characteristics and clinical features of the subjects enrolled are shown in Table 1.

**Table 1:** Characteristics of primary Sjögren’s syndrome (pSS) patients, Sicca, and health controls.

	pSS (*n* = 31)	Sicca (*n* = 7)	Health controls (*n* = 31)
Age, mean ± SD	49.4 ± 10	44 ± 18.6	46.2 ± 8
Female sex (%)	93.55	100	90.32
Negative anti-SSA and anti-SSB (%)	6 (19.35%)	6 (85.71%)	–
Positive anti-SSA (%)	11 (35.48%)	1 (14.28%)	–
Positive anti-SSA and anti-SSB (%)	14 (45.16%)	0	–
ESSDAI, mean ± SD (range)	5.85 ± 5.78 (0–21)	–	–

*ESSDAI, EULAR Sjögren’s Syndrome Disease Activity Index*.

*The pSS patients were diagnosed according to the 2002 American-European Consensus Group criteria without treatment*.

### Determination of Percentages of Treg Cells and Th17 Cells in Peripheral Blood

PBMCs were isolated from the 10 pSS patients or 10 healthy subjects using density-gradient centrifugation and stimulated for 4 h with PMA, ionomycin, and Golgi-plug (BD Bioscience). Cells were stained with anti-CD4-PerCP (clone RPA-T4, BD Biosciences), anti-CD25-PE (Clone M-A251, BD Biosciences), and anti-CD127-FITC (Clone HIL-7R-M21, BD Biosciences). IL-17A and FOXP3 expression were determined by intracellular staining with anti-IL-17A-PE (clone eBio64DEC17, eBioscience) and anti-FOXP3-PerCP-Cy5.5 (clone 236A/E7, BD Biosciences). Events were collected with a FACS Verse flow cytometer (BD Biosciences, NJ, USA) and analyzed with Flow Jo software (Tree Star, Ore).

### Differentiation of Th17 Cells From CD4^+^ Naïve T Cells

PBMCs were isolated from healthy subjects using density-gradient centrifugation, and CD4^+^ naïve T cells were enriched from PBMCs using human naïve CD4^+^ T cell isolation kit II (Miltenyi Biotec, Germany). For *in vitro* differentiation, isolated human naïve CD4^+^ T cells were stimulated with plate-bound human anti-CD3/CD28 (clone OKT-3 and clone 9.3 Bio X Cell, respectively, 5 µg/ml of each) and cultured with IL-6 (50 ng/ml), TGF-β1 (0.5 ng/ml), IL-1β and IL-23 (both 10 ng/ml), anti-IFN-γ and anti-IL-4 (10 µg/ml for each, Bio X Cell), with or without 10 ng/ml IL-2 for 8 days in complete RPMI 1640 medium. Cells were incubated with 5 µM STAT5 inhibitor (STAT5-IN-1, MedChem Express) 1 h prior to IL-2 stimulation. All cytokines were purchased from R&D systems, except for IL-2 from PeproTech.

### Quantitative Real-Time PCR

Total RNAs were isolated from minor salivary glands (MSGs) biopsy tissues, PBMCs or differentiated Th17 cells using TRIzol reagent (Life Invitrogen, Carlsbad, CA, USA). cDNAs were reverse transcribed from 0.1 µg total mRNA using the High Capacity cDNA Reverse Transcription Kit (Applied Biosystems, USA). For sample analysis, the threshold was set based on the exponential phase of amplifications, and CT value for samples was determined. The resulting data were analyzed with the comparative CT method for relative gene expression quantification against house keeping gene *GAPDH*. qPCR was performed using the Bio-Rad SYBR Green intercalating fluorophore system. Primers were listed in the Table S1 in Supplementary Material.

### Immunohistochemical Staining

Human MSG tissues from 10 pSS patients and 7 Sicca control patients were obtained after informed consent. The gland tissues were fixed with 4% paraformaldehyde, followed with embedding in paraffin blocks. The slides were heated at 65°C for 30 min, followed by paraffin removal with xylene and subsequent rehydration with ethanol. Antigen retrieval was performed in citrate buffer (pH 6.0) at a sub-boiling temperature for 20 min. Samples were blocked with 10% goat serum for 1 h at RT and incubated with primary antibody (1:100) overnight at 4°C. The slides were visualized using streptavidin peroxidase IHC assay kit (ZSGB-bio, China) and counter stained with hematoxylin. The primary antibody of immunohistochemistry was performed using anti-IL-17A (Clone G-4, Santa Cruz), anti-CD4 (Clone EPR6855, Abcam), anti-p-STAT3 (Clone EP2147Y, Abcam)m and anti-p-STAT5 (Clone E208, Abcam). Images were obtained using an OLYMPUS-BX51 microscope at 10 × 10 or 40 × 10 magnification.

### p-STAT3 and p-STAT5 Determination by Cytometric Analysis

The levels of p-STAT3 and p-STAT5 were determined by cytometric analysis. In brief, PBMCs were isolated from pSS patients or healthy controls using density-gradient centrifugation. PBMCs were washed and fixed for 15 min at room temperature. Cells were stained with anti-CD4 (Clone L200, BD Biosciences), anti-p-STAT3 (Clone D3A7, Cell Signaling), and anti-p-STAT5 (Clone Y694, Cell Signaling) using BD Phosflow kit according to the manufacturer’s instructions. The median fluorescence intensities were analyzed for p-STAT3 and p-STAT5 levels.

### Immunofluorescence and Confocal Analysis

Standard immunofluorescence analysis was performed on MSG paraffin-embedded sections to assess whether IL-17A co-localized with CD4 cells. The primary antibodies were anti-IL-17A (Clone G-4, Santa Cruz), anti-CD4 (Clone EPR6855, Abcam) and then stained with FITC- or Cy3-conjugated anti-mouse or anti-rabbit Abs and nuclei were labeled with DAPI. Confocal analysis was used to acquire fluorescence imaging.

### Chromatin-Immunoprecipitation Assay

Human naïve CD4^+^ T cells were differentiated under Th17 differentiation conditions for 8 days, followed by cross-linking for 8 min with 1% (vol/vol) formaldehyde. Cells were collected and lysed by sonication. Cell lysates were immunoprecipitated with anti-H3K4me3 (ab8580, Abcam), anti-STAT5 antibody (#9363, Cell Signaling), and anti-STAT3 antibody (clone c-20, Santa Cruz). After washing and elution, crosslinks were reversed for 4 h at 65°C. The eluted DNA was purified, and analyzed by qPCR with custom-designed primers, Forward: TAGCACCAACAGCACTTCTAGC Reverse: TCAGCACATGCATCATTGTCAG using a Bio-Rad SYBR Green intercalating fluorophore system. The Ct value for each sample was normalized to corresponding input value.

### ELISA

The serum levels of IL-2, IL-6, and IL-17A were quantified with commercial enzyme-linked immunosorbent assay kits (Dakewei Biotech Company Ltd., Shenzhen, China), following the manufacturer’s instructions.

### Statistical Analysis

Experimental values were expressed as mean ± SEM, and the differences in means were analyzed using Student’s *t*-test. Data from three different groups were analyzed by using one-way ANOVA. The Mann–Whitney *U* test and Spearman’s correlation analysis were used. *p* Value <0.05 was considered statistically significant.

## Results

### Increased Infiltration of Th17 Cells but Unaffected Treg Cells in pSS Patients

**Figure 1:**
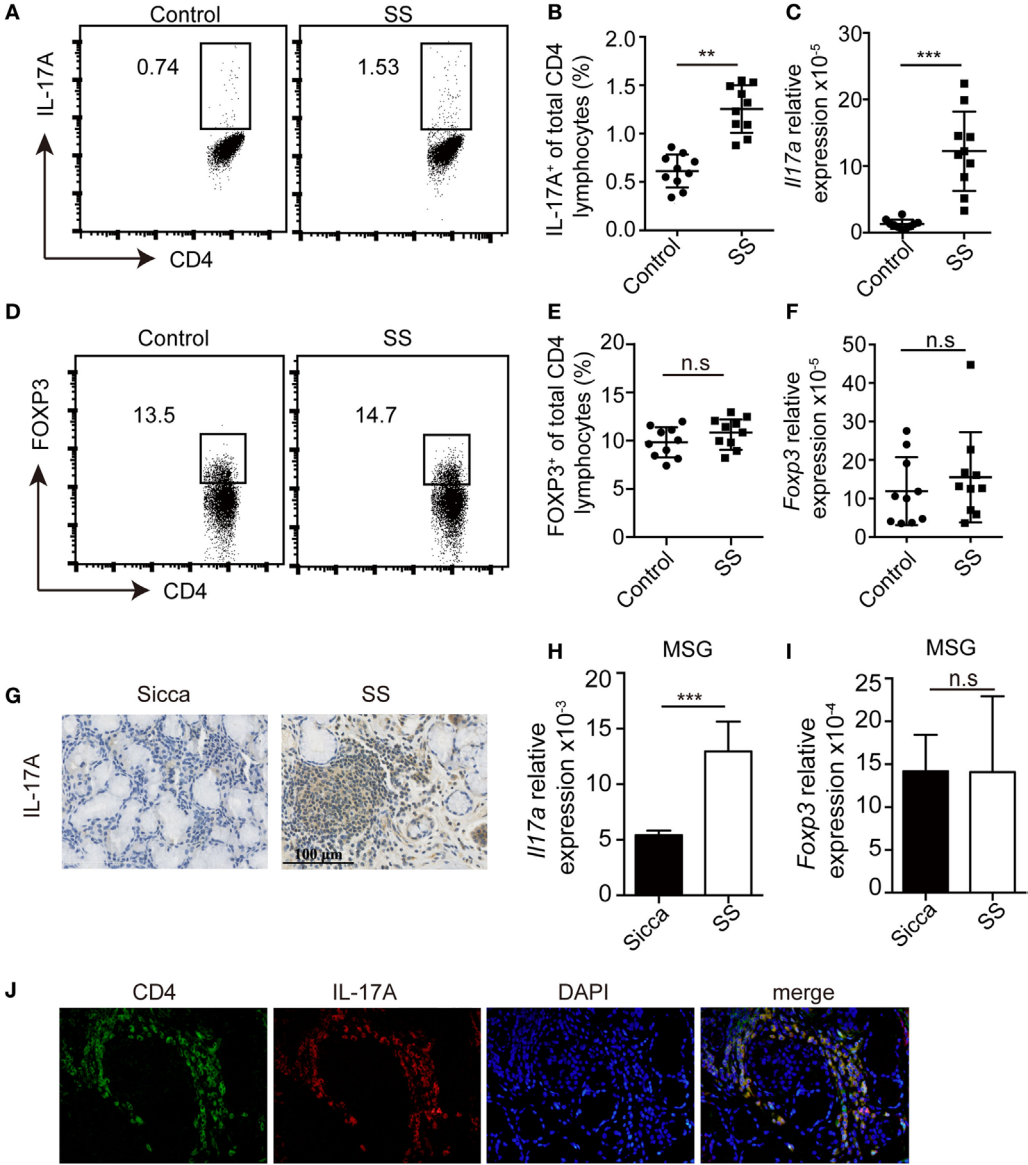
Increased Th17 cells but no change of Treg cells in primary Sjögren’s syndrome (pSS) patients. **(A,B)** The percentages of IL-17^+^CD4^+^ T cells in the PBMCs from healthy donors or pSS patients were determined by flow cytometry. Representative plot **(A)** and histogram analysis **(B)** were shown. **(C)** The amount of *Il17a* mRNA in PBMCs was determined by q-PCR. **(D,E)** The percentages of Foxp3^+^CD4^+^ T cells in the PBMCs were determined by flow cytometry. Representative plot **(D)** and histogram analysis **(E)** were shown. **(F)** The amount of *Foxp3* mRNA in PBMCs from healthy donors or pSS patients was determined by q-PCR. **(G)** IL-17A levels in the minor salivary gland (MSG) from Sicca or pSS patients were determined by immunohistochemistry. **(H–I)** The amounts of *Il17a* **(H)** and *Foxp3* mRNA **(I)** were determined by q-PCR. **(J)** Immunofluorescence staining of CD4 and IL-17A in the MSG specimens from pSS patients. Representative images are shown (400×). **p < 0.01, ***p < 0.001.

To evaluate whether Th17 and Treg cells were dysregulated in pSS patients, we determined the percentage of Th17 and Treg cells in the periphery. Compared with healthy donors (*n* = 10), the percentages of Th17 cells were significantly higher in pSS patients (*n* = 10) (Figures 1A,B). Consistent with this, *Il17a* mRNA was also increased in the PBMCs from pSS patients (Figure 1C). By contrast, we found no difference in the percentage of Treg cells, which were defined by CD4^+^FOXP3^+^ or CD4^+^CD25^+^CD127^−^ in the periphery between pSS and healthy subjects (Figures 1D,E; Figure S1 in Supplementary Material). The mRNA of *Foxp3* in PBMCs was also comparable (Figure 1F). Furthermore, we detected the expression of IL-17A in the MSG tissue. While IL-17A was barely detected in the MSG of Sicca controls, its expression was enhanced in pSS patients (Figure 1G). Accordingly, we found increased expression of *Il17a* mRNA but not *Foxp3* in the MSG of pSS patients (Figures 1H,I). Immunofluorescence staining showed co-localization of CD4 and IL-17A in the MSG of pSS patients (Figure 1J). Taken together, all of these data demonstrate an increased infiltration of Th17 cells and unaltered Treg cells presence in pSS patients.

### The Decreased IL-2 and Increased IL-6 Levels in pSS Patients

**Figure 2:**
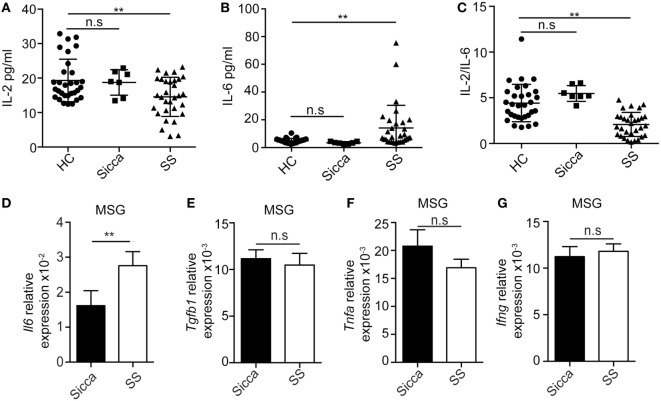
Enhanced IL-6 but reduced IL-2 expression in primary Sjögren’s syndrome (pSS) patients. **(A–C)** Serum levels of IL-2 **(A)**, IL-6 **(B)**, and IL-2/IL-6 **(C)** were determined by ELISA in the healthy subjects (*n* = 31), Sicca (*n* = 7), and pSS patients (*n* = 31). **(D–G)** Expressions of *Il6* **(D)**, *Tgfb1* **(E)**, *Tnfa* **(F)**, and *Ifng* **(G)** mRNAs in the minor salivary gland (MSG) from either Sicca patients or pSS patients were determined by q-PCR. **p < 0.01.

Next we compared the levels of IL-2, IL-6, and IL-17A in the plasma of healthy donors, Sicca, and pSS patients. While the levels of IL-2 in health controls and Sicca patients were comparable, it was significantly lower in pSS patients (Figure 2A). By contrast, the circulating amounts of IL-6 and IL-17A were significantly enhanced in pSS patients (Figure 2B; Figure S2A in Supplementary Material). In addition, the ratio of IL-2 and IL-6 was significantly decreased in pSS patients (Figure 2C). Furthermore, there was a negative correlation between the level of IL-2 and the level of IL-17A in pSS patients (Figure S2B in Supplementary Material). *Il6* mRNA expression in MSG from pSS patients were significantly higher compared with Sicca patients (Figure 2D). However, the expression of *Tgfb1*, a cytokine chiefly involved in Treg and IL-6 dependent Th17 differentiation, was comparable in MSG between Sicca and pSS patients (Figure 2E). Furthermore, mRNAs of other pro-inflammatory cytokines *Tnfa* and *Ifng* were also comparable (Figures 2F,G). These data demonstrate that the expression of IL-2 and IL-6 are dysregulated, which may corroborate with the increased infiltration of Th17 cells in pSS patients.

### IL-2 Inhibits Human Th17 Differentiation Independently of the Induction of Tregs

**Figure 3:**
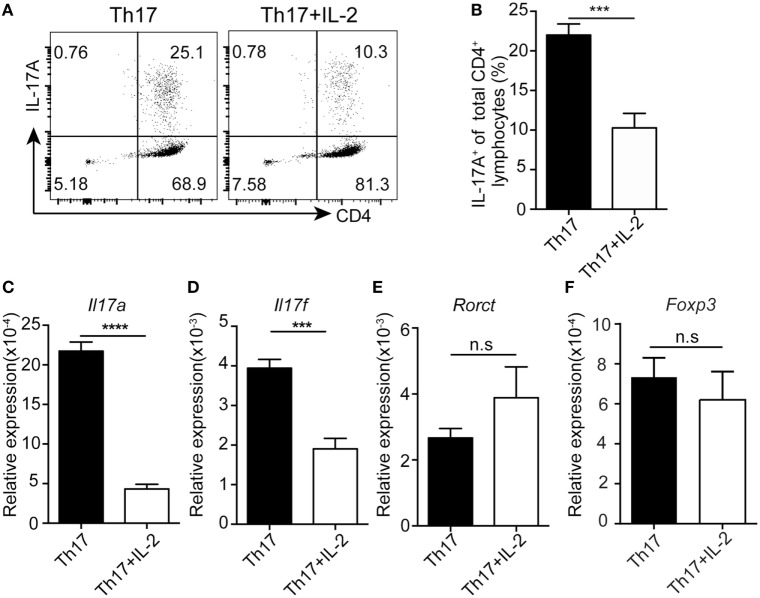
IL-2 inhibits human Th17 differentiation without alternation of FOXP3 expression. Naïve CD4^+^ T cells were stimulated under Th17 conditions with or without IL-2 for 8 days. The percentages of CD4^+^IL-17A^+^ cells were determined by intracellular staining **(A)**. Histogram analysis of the percentages of the differentiated Th17 cells treated with or without IL-2 **(B)**. mRNA expressions of *Il17a* **(C)**, *Il17f* **(D)**, *Rorct* **(E)**, and *Foxp3* **(F)** genes was assessed by q-PCR in differentiated Th17 cells treated with or without IL-2. ***p < 0.001.

To test whether IL-2 directly inhibits human Th17 differentiation, we differentiated human PBMCs into Th17 cells *in vitro* with or without IL-2. Treatment of IL-2 decreased the number of human Th17 differentiation (Figures 3A,B), while the expression of FOXP3 was not changed by adding IL-2 under the Th17 differentiation, suggesting that a FOXP3-independent suppression of Th17 differentiation by IL-2. Furthermore, qPCR results showed that *Il17a* and *Ill17f* were significantly decreased after IL-2 treatment while *Rorc*t and *Foxp3* mRNA expression were not affected (Figures 3C–F), indicative of a direct suppression of *Il17* but not *Rorc*t gene expression by IL-2. Together, these data suggest that IL-2 could directly inhibit Th17 differentiation in a cell intrinsic manner, not through the induction of FOXP3-expressing Treg cells, which can inhibit Th17 generation in trans.

### Competitive Binding of STAT5 and STAT3 to the Human *Il17a* Locus

**Figure 4:**
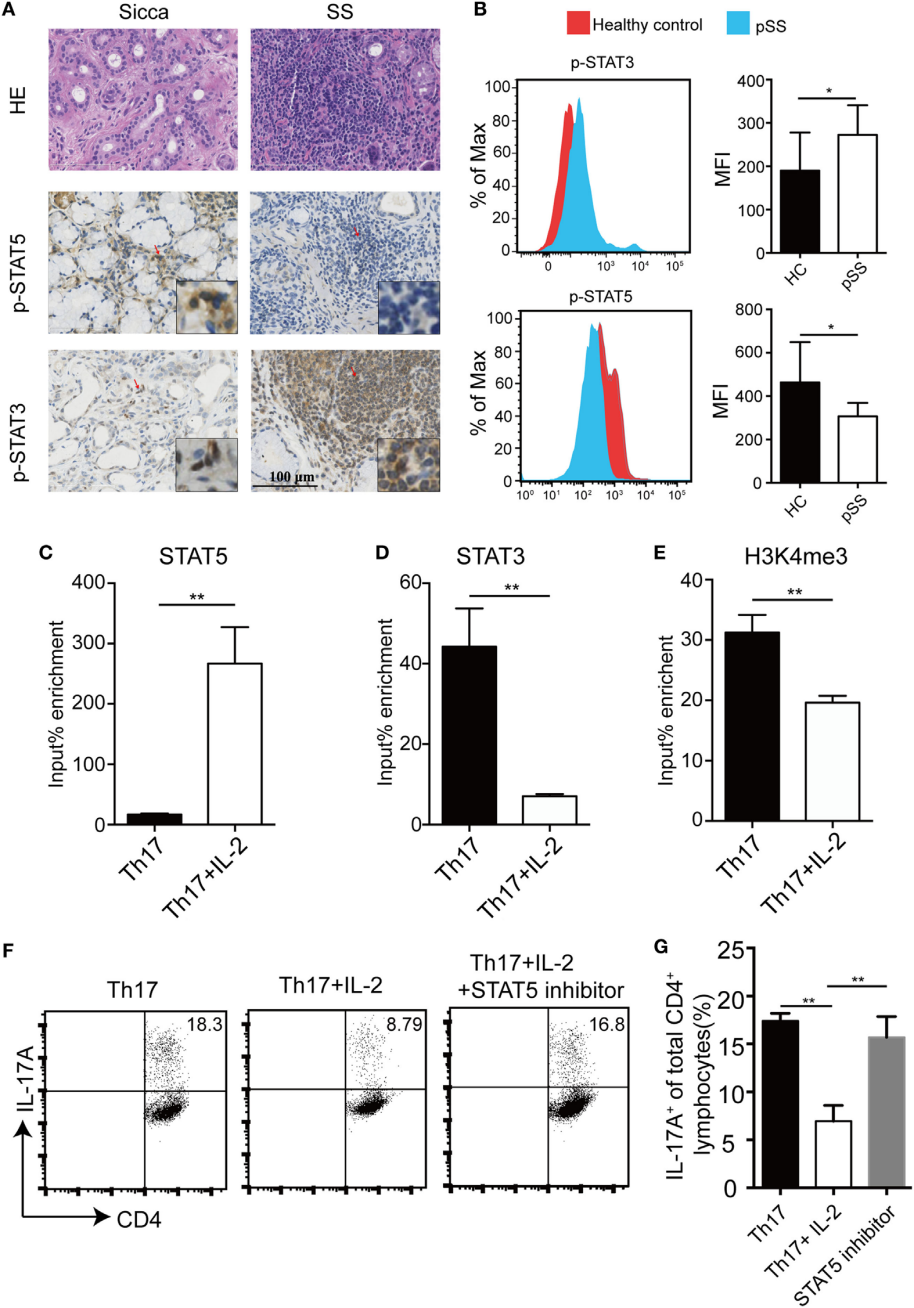
IL-2-induced STAT5 binds to STAT3-binding site in human *Il17a* locus. **(A)** H&E and IHC staining with anti-p-STAT3 and anti-p-STAT5 antibody on the minor salivary gland (MSG) tissues from 10 primary Sjögren’s syndrome (pSS) patients and 7 Sicca patients. Representative images are shown. **(B)** The levels of p-STAT3 and p-STAT5 in CD4^+^ T cells in the PBMCs from healthy donors (*n* = 5) or pSS (*n* = 5) patients were determined by flow cytometry. Representative plot (left) and histogram analysis (right) were shown. **(C–E)** Naïve CD4^+^ T cells were stimulated under Th17 conditions with or without IL-2, then crosslinked, and immunoprecipitated with anti-STAT5 **(C)**, anti-STAT3 **(D)**, and anti-H3K4me3 **(E)**. Immunoprecipitated DNA was amplified by q-PCR and expressed as a percentage to input DNA. **(F,G)** Human naïve CD4^+^ T cells were differentiated under Th17 conditions with IL-2 by adding STAT5 inhibitor STAT5-IN-1 (5 µM) for 8 days. The percentages of CD4^+^IL-17A^+^ cells were determined by intracellular staining **(F)**. Histogram analysis of the percentages of the differentiated Th17 cells treated with or without STAT5 inhibitor **(G)**. *P* value was determined with unpaired *t*-test. Data are pooled from two independent experiments (error bars denote SEM). *p < 0.05, **p < 0.01.

IL-6 induces STAT3 activation which mediates its most biological functions, whereas IL-2 signals through STAT5 phosphorylation. Next, we detected the levels of p-STAT3 and p-STAT5 in pSS SGs and controls using immunohistochemistry. While p-STAT5 levels were reduced in the MSG of pSS patients compared with Sicca patients, p-STAT3 levels were higher (Figure 4A). This is consistent with our other finding that p-STAT3 levels were higher while p-STAT5 levels were reduced in peripheral CD4^+^ T cells of pSS patients, compared with healthy controls (Figure 4B). To further investigate the relationship between STAT3 and STAT5 in pSS patients, we analyzed the binding of STAT3 and STAT5 to the *Il17a* promoter region after IL-2 treatment in the Th17 differentiation condition. We found IL-2 induced enhanced STAT5 binding but IL-6-induced STAT3 binding in the same site was diminished, indicating a direct competition between STAT5 and STAT3 for the binding to the *Il17a* locus (Figures 4C,D). Furthermore, adding of IL-2 was associated with a reduction of occupancy of H3K4me3 in *Il17* promoter, a transcriptional activation mark (Figure 4E). To further substantiate the involvement of STAT5 activation in the inhibitory effect of IL-2 on Th17 differentiation, we compared the IL-2 effects on Th17 differentiation in the absence or presence of a STAT5 inhibitor, STAT5-IN-1. We found that addition of the STAT5 inhibitor STAT5-IN-1 abolished the inhibitory effect of IL-2 on Th17 differentiation (Figures 4F,G). Together, these data demonstrate that IL-2 suppresses *Il17a* gene expression *via* activation of STAT5, which competes with STAT3 for the same binding site.

## Discussion

In this study, our data showed that reduced IL-2 expression and increased IL-6 expression in pSS patients, which was associated with an increased infiltration of Th17 cells. However, the number of Treg cells in pSS patients was not changed. These findings highlight the importance of Treg-independent, tonic IL-2 suppression of Th17 differentiation in the pathogenesis of pSS.

Both reduced and unchanged number of Treg cells in pSS patients have been reported (18, 19). The discrepancy for this is currently unclear. Furthermore, the suppressive function of Treg is also intact in pSS patients (19). In line with this, we found no difference of TGF-β expression and numbers of FOXP3^+^ Treg cells in pSS patients. These suggest that beyond Treg cells, the development of pSS may be caused by defects of other pathways which mediate self-tolerance for autoimmunity.

Th17-associated cytokines including IL-6, IL-21, and IL-22 are reported to be elevated in pSS patients (29, 30). Consistent with this, we also found increased IL-6 expression in pSS. The polymorphisms of *Il2-Il21r* region are implicated in multiple autoimmune diseases (31). Our study demonstrates a reduction of circulatory IL-2 in pSS patients and accompanied with enhanced Th17 generation, which is consistent with previous reports that reduced IL-2 availability in SLE and type I diabetes patients (32, 33). These data suggest that the inadequacy of IL-2 may be more ubiquitous in autoimmune diseases and more attention should be paid to the ratio of IL-2:IL-6 in the pathogenesis of autoimmune diseases.

While IL-2 mediates T cell proliferation and memory T cell generation, IL-2 also promotes Treg generation and limits Th17 and Tfh differentiation, thus playing critical role in mediating tolerance (11, 12, 34, 35). For limiting Th17, IL-2-induced STAT5 activation competes with STAT3 in *Il17a/f* loci in mice and similar mechanism exists in regulation of *Bcl-6* in Tfh cells (13, 36). Although IL-2 suppressed *Il17* expression, IL-2 did not affect the expression of *Rorct* and *Foxp3* under Th17 differentiation condition (Figures 3C–F) and addition of a STAT5 inhibitor abolished the inhibitory effect of IL-2 on human Th17 differentiation (Figures 4F,G), indicating a direct effect of STAT5 on *Il17* locus. In support of this, IL-2-induced STAT5 directly bound to the same binding site as IL-6-induced STAT3 in *Il17* locus in human primary T cells, indicating a similar regulation of Th17 differentiation by IL-2 in the two species.

Fine-tuning IL-2 signaling pathways has received great attention in the treatment of cancer and autoimmune diseases (21, 24). For example, low dose IL-2 has been clinically beneficial in lupus and type I diabetes (20, 21, 37). While most of the studies focus on the role of IL-2 in expansion of Treg cells, other types of T cells and NK cells are also responsive to high-doses of IL-2 (24, 25). The toxicity of IL-2 remains a hinder for wide use of IL-2 in clinic (28). The sensible IL-2 administration to avoid inappropriate immune responses is critical. Our study uncovered that the IL-2 signaling under steady state is critical for direct inhibition of Th17 differentiation, without the participation of Treg cells, in preventing autoimmunity. While our study does not dispute the role of IL-2 to expand Treg cells, it is of note that IL-2 can directly inhibit Th17 differentiation *via* deploying STAT5 to compete with STAT3.

In conclusion, our study demonstrated that Th17 cells are enhanced, whereas Treg cells are hardly regulated in pSS. This increased Th17 differentiation is largely due to a deficiency of IL-2, which could directly suppress Th17 differentiation. This study provides further understanding of IL-2-mediated therapeutic application in autoimmune diseases.

## Ethics Statement

Full name of the ethics committee is Tongji Hospital, Tongji Medical College, Huazhong University of Science, and Technology Institutional Review Board Approval. The ethics IRB ID is: TJ-C20151109.

## Author Contributions

LD and X-PY designed the study. CZ, XD, PL, ZW, YX, JL, and BM performed the experiments. KJ, HY, WM, ZL, and HL analysed and interpretated the data. JL and BM wrote the manuscript. All the authors read and approved the final manuscript.

## Conflict of Interest Statement

The authors declare that the research was conducted in the absence of any commercial or financial relationships that could be construed as a potential conflict of interest.
