# Supplementary material for: IL-2 Inhibition of Th17 Generation Rather Than Induction of Treg Cells Is Impaired in Primary Sjögren’s Syndrome Patients
Source: Front Immunol. 2018 Aug 13;9:1755. doi: 10.3389/fimmu.2018.01755 (PMC6100298; doi:10.3389/fimmu.2018.01755)
Supplement: Supplementary file 1 [file Table_1.doc]

**Supplementary Table 1. Primers sequence are as follows:**

| **Primer** | **Forward** (5’-3’) | **Reverse** (5’-3’) |
| --- | --- | --- |
| *h-gapdh* | GGAGCGAGATCCCTCCAAAAT | GGCTGTTGTCATACTTCTCATGG |
| *h-Il-6* | ACTCACCTCTTCAGAACGAATTG | CCATCTTTGGAAGGTTCAGGTTG |
| *h-Foxp3* | GTGGCCCGGATGTGAGAAG | GGAGCCCTTGTCGGATGATG |
| *h-Tgfβ1* | GGCCAGATCCTGTCCAAGC | GTGGGTTTCCACCATTAGCAC |
| *h-Tnf-α* | CCTCTCTCTAATCAGCCCTCTG | GAGGACCTGGGAGTAGATGAG |
| *h-Il17a* | TCCCACGAAATCCAGGATGC | GGATGTTCAGGTTGACCATCAC |
| *h-Ifng* | CCAACTAGGCAGCCAACCTAA | AGCACTGGCTCAGATTGCAG |


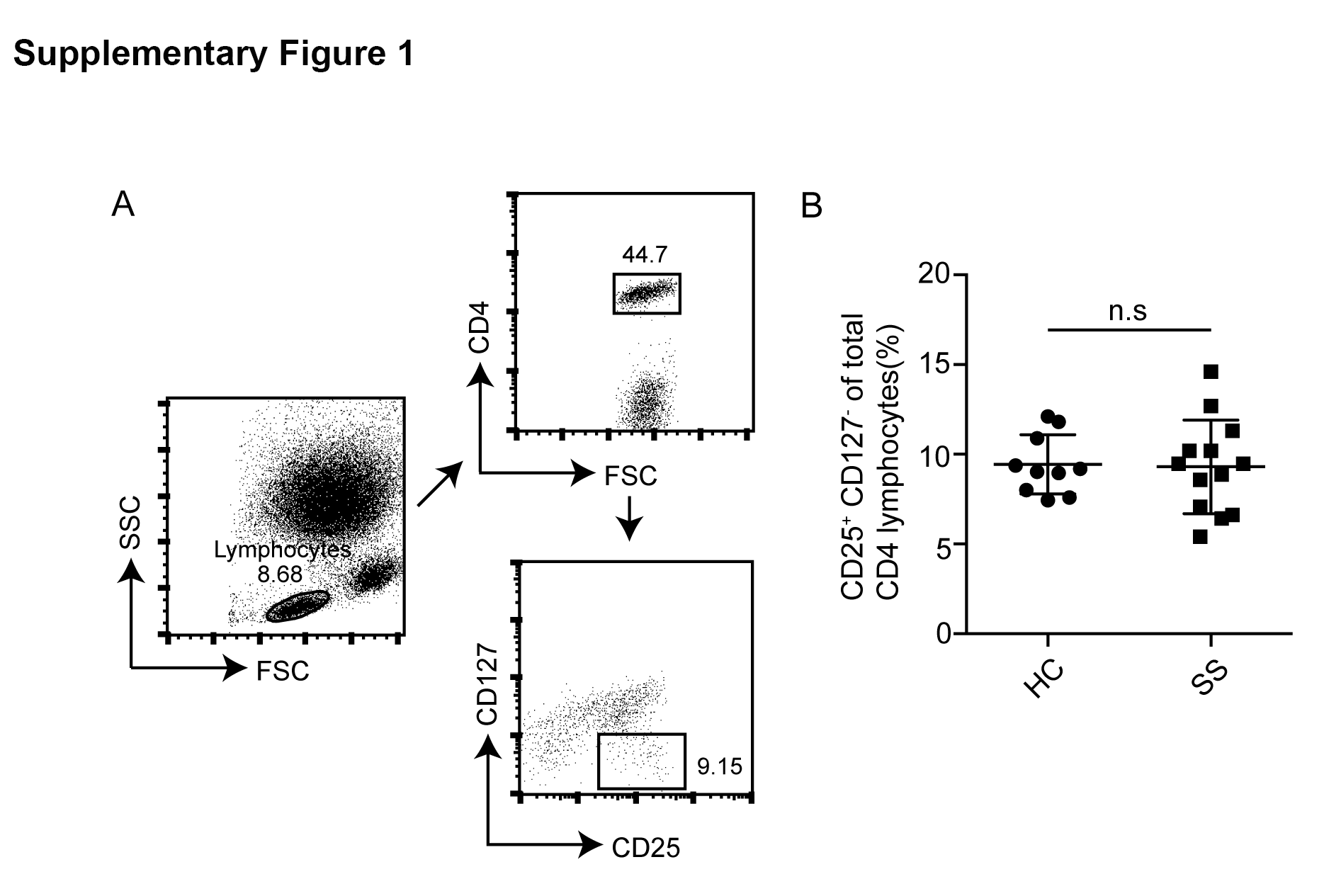


**Supplementary Figure 1. The percentages of CD4^+^CD25^+^CD127^-^ Treg cells are comparable between pSS patients and controls.**

(A-B). The percentages of CD4^+^CD25^+^CD127^-^ Treg cells in PBMCs from pSS patients (n=13) or healthy controls (n=10) were determined by flow cytometry. Gating strategy of human CD4^+^CD25^+^CD127^-^Treg cells (A) and histogram analysis (B) were shown.


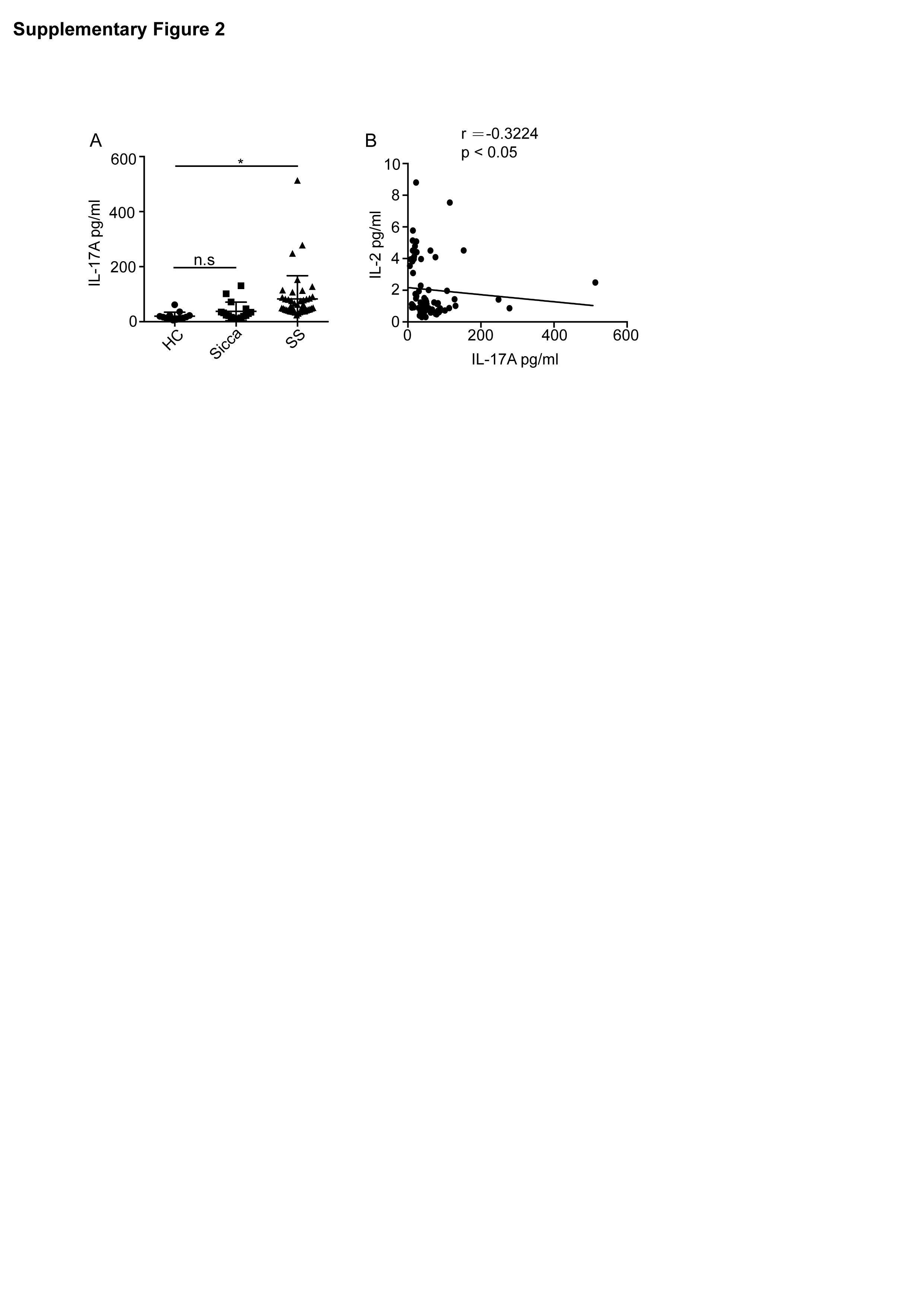


**Supplementary Figure 2. Circulating levels of IL-17A are enhanced and negatively correlated with the levels of IL-2 in pSS patients.**

(A) Serum levels of IL-17A were determined by ELISA in the healthy subjects (n=13), Sicca (n=17) and pSS patients (n=43). (B) Correlation analysis of IL-2 and IL-17A was determined by Spearman’s analysis. **p*<0.05.
